# Determination of Dy substitution site in Nd_2−x_Dy_x_Fe_14_B by HAADF-STEM and illustration of magnetic anisotropy of “g” and “f” sites, before and after substitution

**DOI:** 10.1038/s41598-021-85713-5

**Published:** 2021-03-18

**Authors:** Syed Kamran Haider, Min-Chul Kang, Jisang Hong, Young Soo Kang, Cheol-Woong Yang, Dongsoo Kim

**Affiliations:** 1 Convergence Research Center for Development of Mineral Resources, Korea Institute of Geoscience and Mineral Resources, Daejeon, 34132 South Korea; 2 Powder and Ceramics Division, Korea Institute of Materials Science, Changwon, Gyeongnam 51508 South Korea; 3 Department of Chemistry, Sogang University, 35, Baekbeomro, Mapogu, Seoul, 04107 South Korea; 4 School of Advanced Materials Science and Engineering, Sungkyunkwan University, Suwon, 16419 South Korea; 5 Department of Physics, Pukyong National University, Busan, 48513 South Korea

**Keywords:** Chemistry, Materials science, Nanoscience and technology

## Abstract

Nd_2_Fe_14_B and Nd_2−x_Dy_x_Fe_14_B (x = 0.25, 0.50) particles were prepared by the modified co-precipitation followed by reduction–diffusion process. Bright field scanning transmission electron microscope (BF-STEM) image revealed the formation of Nd–Fe–B trigonal prisms in [− 101] viewing zone axis, confirming the formation of Nd_2_Fe_14_B/Nd_2−x_Dy_x_Fe_14_B. Accurate site for the Dy substitution in Nd_2_Fe_14_B crystal structure was determined as “f” site by using high-angle annular dark field scanning transmission electron microscope (HAADF-STEM). It was found that all the “g” sites are occupied by the Nd, meanwhile Dy occupied only the “f” site. Anti-ferromagnetic coupling at “f” site decreased the magnetic moment values for Nd_1.75_Dy_0.25_Fe_14_B (23.48 μB) and Nd_1.5_Dy_0.5_Fe_14_B (21.03 μB) as compared to Nd_2_Fe_14_B (25.50 μB). Reduction of magnetic moment increased the squareness ratio, coercivity and energy product. Analysis of magnetic anisotropy at constant magnetic field confirmed that “f” site substitution did not change the patterns of the anisotropy. Furthermore, magnetic moment of Nd_2_Fe_14_B, Nd_2−x_Dy_x_Fe_14_B, Nd (“f” site), Nd (“g” site) and Dy (“f” site) was recorded for all angles between 0° and 180°.

## Introduction

Nd_2_Fe_14_B type magnets have the highest recorded maximum energy product (BH)_max_ among permanent magnets^1–5^. They have drawn huge attention due to their wide applications in wind turbines, hybrid-electric vehicles, transducers, magnetic fluids, magnetic elastomers, sensors, magnetic separators, magnetic levitation systems, loudspeakers, generators and motors^6–11^. Current maximum coercivity of unsubstituted Nd_2_Fe_14_B is recorded as 10 kOe, which makes it economically inadequate for commercial purpose. However, magnetic properties of Nd_2_Fe_14_B have been improved significantly by the substitution of Nd with rare earth elements (RE)^12–21^. Hence the synthesis of Nd_2−x_RE_x_Fe_14_B by RE substitution for Nd is vital for the applications of Nd_2_F_e14_B on commercial scale. The site of RE atoms in the Nd_2−x_RE_x_Fe_14_B crystal lattice is very critical for its anisotropy and magnetic moment. Nd_2_Fe_14_B is a tetragonal structure with four formula units and 68 atoms, which has two unequal sites for Nd (RE), “g” site and “f” site. When RE substitutes for Nd in Nd_2_Fe_14_B crystal lattice, it may either substitute at “f” or “g” site. Aftab, Kitagawa and Liu et al. have worked to estimate the substitution site for RE in Nd_2_Fe_14_B theoretically, by using density function theory (DFT)^19–21^. Aftab et al. applied Vienna ab initio simulation package (VASP) with a pseudo-potential and projected-augmented wave (PAW) method. Kitagawa et al. applied the linear combination of pseudo-atomic-orbital (LCPAO) method, while Liu et al. used full potential plane-wave plus muffin-tin orbital method.

Experimental evidences for Dy site in (Nd-Dy)_2_Fe_14_B crystal lattice was provided by Itakura et al.^22^ Although they presented STEM-HAADF image but d-spacing value for the [001] facet deviated from the standard values. Absence of the stoichiometric ratio between Nd:Dy:Fe and distribution of Dy only near grain boundary raised more questions, hence it was interesting for the contemporary researchers to answer them. Saito et al.^23^ used neutron diffraction technique and determined the quantitative distribution of Dy at substitution sites. They postulated that Dy substitutes at both the “f” and “g” sites, and population of Dy on each site depends on the annealing temperature of the experiment. This finding was different from the previous studies, those suggested that Dy only substitutes at “f” site. Furthermore, magnetic anisotropic properties of “f” and “g” sites in Nd_2−x_Dy_x_Fe_14_B were not yet studied. In order to answer the questions discussed above and a comprehensive study of “f” and “g” sites in Nd_2−x_Dy_x_Fe_14_B was required.

In this work, we prepared Nd_2_Fe_14_B and Nd_2−x_Dy_x_Fe_14_B with optimized co-precipitation method followed by reduction–diffusion (R–D) process. This co-precipitation method is similar with the approach reported by Ma and Palaka et al. with minor modification^24,25^. Detail of this modification is explained in the “supplementary information”. Site preference of Dy in Nd_1.5_RE_0.5_Fe_14_B particles was confirmed through the crystal structure determination by using HAADF-STEM. Effect of Dy substitution on the magnetic moment, magneto-crystalline anisotropy energy and coercivity of the Nd_2−x_Dy_x_Fe_14_B particles is also studied. Nd_2−x_Dy_x_Fe_14_B sample was rotated in the rotating angle range of 0° to 180° at the constant magnetic field to observe variation of anisotropic patterns after the substitution. Effect of the substitution on the anisotropic properties of “f” and “g” sites in the Nd_2−x_Dy_x_Fe_14_B crystal is also studied.

## Methods

### Materials

All chemicals used in this work, including neodymium (III) chloride hexahydrate (NdCl_3_·6H_2_O), iron (III) chloride hexahydrate (FeCl_3_·6H_2_O), sodium hydroxide (NaOH), boric acid (H_3_BO_3_), calcium hydride (CaH_2_), dysprosium (III) chloride hexahydrate (DyCl_3_·6H_2_O), ethyl alcohol (C_2_H_5_OH) and acetone (CH_3_COCH_3_) were analytical grade and obtained from Sigma-Aldrich Co.

### Preparation of Nd_1.5_RE_0.5_Fe_14_B particles

Schematics for the preparation procedure is shown as the Fig. S1 and detailed process of synthesis is explained in the “supplementary information”. In brief, all the metal chlorides of Nd, Fe and Dy were dissolved in deionized (D.I.) water under the stirring to obtain the clear solution. NaOH (3.5 M) was added to the solution in a drop wise until the pH approached to 13. The resultant solution was stirred continuously for 4 h. Then, products were washed twice with D.I. water and ethanol, and dried overnight at 353 K (80 °C) followed by annealing at 973.15 K (700 °C) for 30 min to convert all hydroxides to oxides. The product was mixed with boric acid and CaH_2_ in a glove box and then pressed into pellet form. The pellet was undergone to R–D via annealing at 1273.15 K (1000 °C) for 3 h with Ar flowing in the furnace. The pellet after R–D was pulverized and washed with water to remove calcium oxide (CaO) completely and rinsed with acetone. To obtain Nd_2_Fe_14_B, weight ratio of Nd:Fe:B was kept as 15:77:8, as per standard ratio for the synthesis of Nd_2_Fe_14_B by R–D. In order to produce Nd_1.75_Dy_0.25_Fe_14_B and Nd_1.5_RE_0.5_Fe_14_B, Nd:Dy:Fe:B ratio was kept as 13.12:1.88:77:8 and 11.25:3.75:77:8, hence 12.5% and 25% of Nd was substituted with Dy, respectively. Nd:Dy ratios in Nd_1.75_Dy_0.25_Fe_14_B and Nd_1.5_Dy_0.5_Fe_14_B were determined as 7.17:1 and 3.13:1 experimentally (Figs. S3, S4). These values were quite close to the theoretically expected ratios of Nd:Dy ratios, 7:1 and 3:1.

### Samples preparation for SEM and TEM

Nd_2_Fe_14_B and Nd_2−x_Dy_x_Fe_14_B powder samples were placed on the movable lower ram and then the prepared solder pieces were placed on the sample. Electrically conductive polymer was poured to encapsulate the solder and powder samples, then heated at 180 °C for 6 min and then pressed under the pressure of 30 kN. Prepared sample was mounted on SEM holder, then mechanically polished with SiC paper, diamond suspension and colloidal silica, subsequently characterized with FE-SEM.

Specimens for TEM (transmission electron microscope) were prepared by focused ion beam (FIB-NX2000, Hitachi) using the lift-out technique. For TEM measurement, sample was treated as the same process reported by Kim et al.^26^ and orientation of sample along [100] zone axis was confirmed by using electron backscatter diffraction (EBSD) (TEAM™ Pegasus, Ametek Co. Ltd. USA).

### Characterization

Crystal structure and phases were determined by X-ray diffraction (XRD) patterns using a Rigaku Diffractometer (XRD, Rigaku). The morphology, size and elemental distribution were observed with field emission scanning electron microscope (JSM-7000F, JEOL), conventional transmission electron microscopy (TEM, JEM-2100F) and aberration corrected TEM (ARM-200F) with energy dispersive X-ray spectroscopy (EDS). TEM was operated at the accelerating voltage of 200 kV. Angular dependent magnetic properties were measured by magnetic property measurement system (MPMS3-Evercool) equipped with rotator. Magnetic properties (M-H curves) of final product were measured by Physical Property Measurement System (PPMS, Evercool II–9 T) in the vibrating sample magnetometer mode. Magnetic field in the range of 7179.3 to − 7179 kA/m was applied to measure the magnetic properties of the final products. JEOL JEM-ARM200F Cs-corrected TEM was used to obtain HAADF-STEM images.

### Numerical method

Full potential linearized augmented plane wave method, as implemented in the Wien2k code, was used to calculate the magnetic moment of Nd_2_Fe_14_B and Nd_2−x_Dy_x_Fe_14_B. Details are described in “supplementary information”.

## Results and discussion

**Figure 1:**
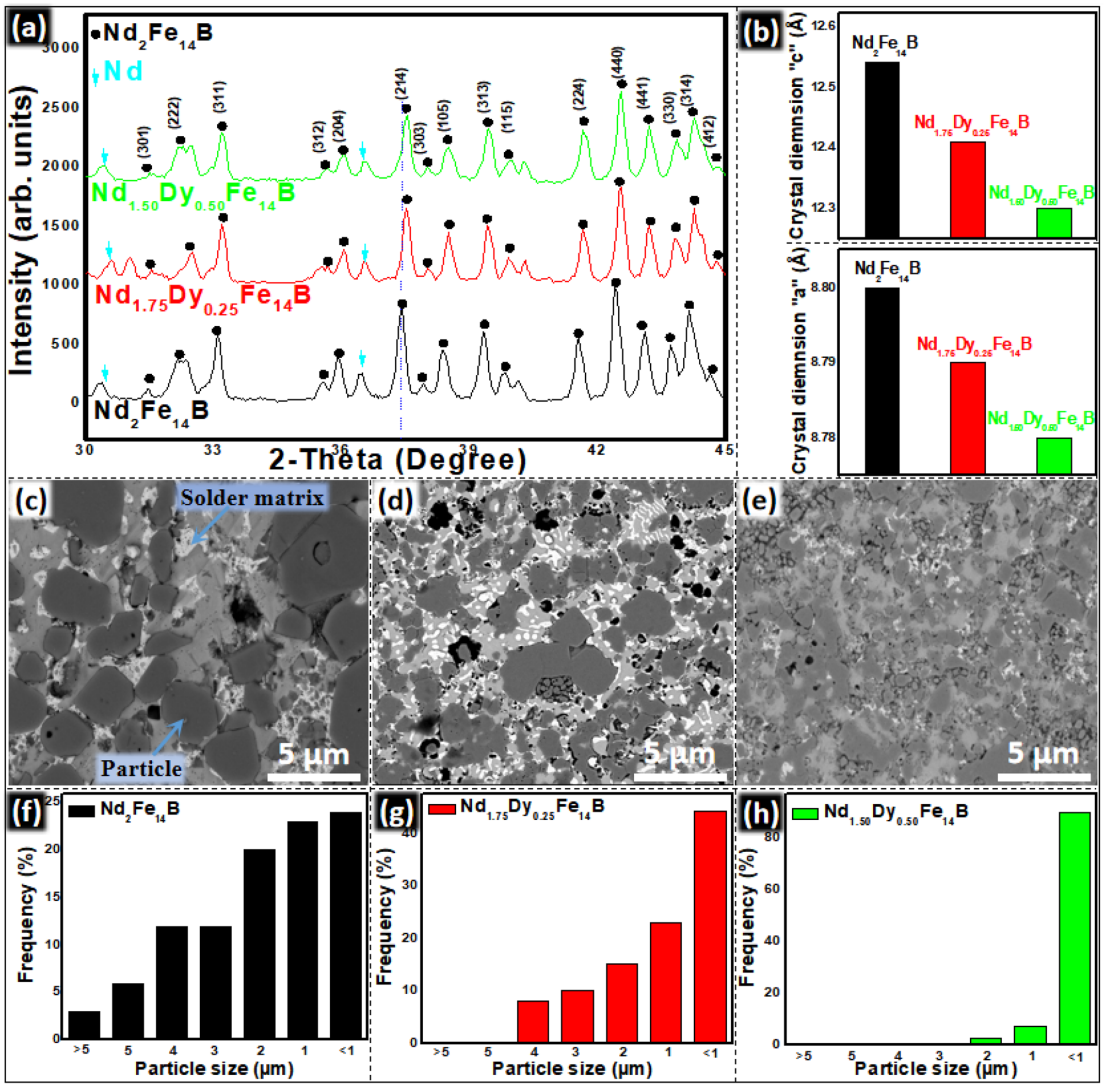
(**a**) XRD patterns of Nd_2_Fe_14_B and Nd_2−x_Dy_x_Fe_14_B particles (**b**) crystal parameters “a” and “c” of Nd_2_Fe_14_B and Nd_2−x_Dy_x_Fe_14_B. BSE-SEM images of (**c**) Nd_2_Fe_14_B (**d**) Nd_1.75_Dy_0.25_Fe_14_B and (**e**) Nd_1.5_Dy_0.5_Fe_14_B particles. Particle size distribution of (**f**) Nd_2_Fe_14_B (**g**) Nd_1.75_Dy_0.25_Fe_14_B and (**h**) Nd_1.5_Dy_0.5_Fe_14_B particles.

Nd_2_Fe_14_B and Nd_2−x_Dy_x_Fe_14_B magnetic particle were prepared by co-precipitation followed by reduction diffusion process. Chemical reactions and mechanisms during co-precipitation and R–D processes are explained in the “supporting information”. R–D reaction follows the mechanism proposed by the Haider et al.^27^ XRD patterns for Nd_2_Fe_14_B and Nd_2−x_Dy_x_Fe_14_B particles are similar due to the almost same crystal structure (Fig. 1a). They have the Nd_2_Fe_14_B (JCPDS #36-1296) as main phase with additional peaks corresponding to the extra Nd phase. Nd substitution with Dy makes the peaks position be shifted to the right side (Fig. 1a).

This is due to the different crystal lattice parameters of Nd_2−x_Dy_x_Fe_14_B and to the smaller ionic radii of Dy (178 pm) as compared to the Nd (181 pm). Both “a “and “c” dimensions of the crystal lattice were decreased after the Dy substitution in the Nd_2_Fe_14_B crystal lattice (Fig. 1b). The decreased values of these Nd_1.5_RE_0.5_Fe_14_B crystal parameters are also evidence of Dy substitution in Nd_2_Fe_14_B crystal. In order to calculate the lattice parameters (“a” and “c”), at first d-spacing values were calculated from the XRD patterns (Fig. 1). h, k and l values were determined from Nd_2_Fe_14_B JCPDS #36-1296. Finally, “c” and “a” value were calculated by the following equation as the same method reported by Rahimi^17^, Cullity^28^, and Charbel^29^, et al.

**Figure 2:**
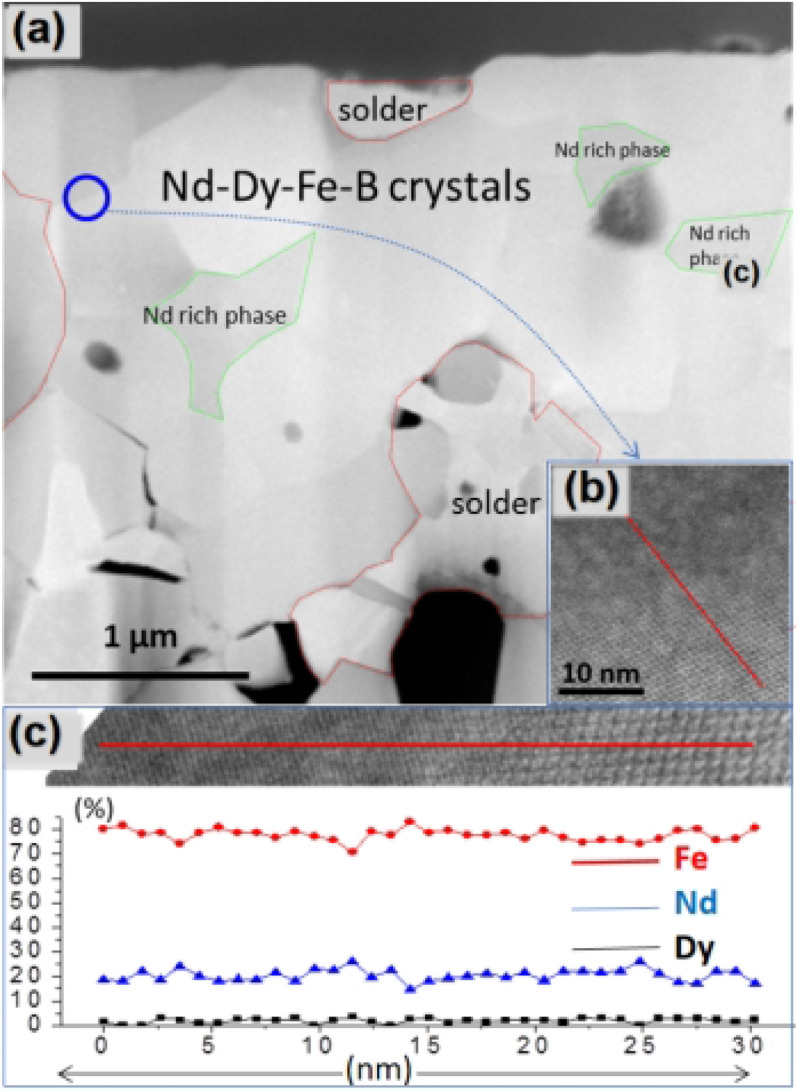
(**a**) LAADF-STEM image of Nd_1.5_Dy_0.5_Fe_14_B, (**b**) HAADF-STEM image and corresponding EDS line profile taken from blue circle in (**a**). (**c**) EDS line profile of (**b**).

SEM-BSE images in Fig. 1c–e revealed that the particles were in irregular shape and the size distribution is in the range of 0.3–10 μm. The Nd_2_Fe_14_B had the largest average particle size as 3.5 μm while Nd_1.5_Dy_0.5_Fe_14_B had the least average particle size as 0.8 μm. Nd_1.75_Dy_0.25_Fe_14_B had the average particle size of 1.7 μm. SEM–EDS confirmed that Nd and Dy are homogeneously distributed with Fe in the particles (Figs. S5, S6). The microstructure, elemental composition and distribution of Nd_1.5_Dy_0.5_Fe_14_B particles were evaluated with STEM as shown in Fig. 2. The elemental distribution of Nd, Dy, Fe, and O was investigated by using STEM-EDS, which confirmed that the Dy was substituted for Nd in the crystal structure and it was distributed inside the grain. Figure 2b is the line EDS taken from the blue circle of LAADF-STEM image of Nd_1.5_Dy_0.5_Fe_14_B. Figure 2c shows the EDS line profile of interface between two fused Nd_1.5_Dy_0.5_Fe_14_B particles, as marked with the blue circle in Fig. 2a. No oxygen was detected in EDS mapping because boundaries of the particles were not exposed to the water during washing process.

**Figure 3:**
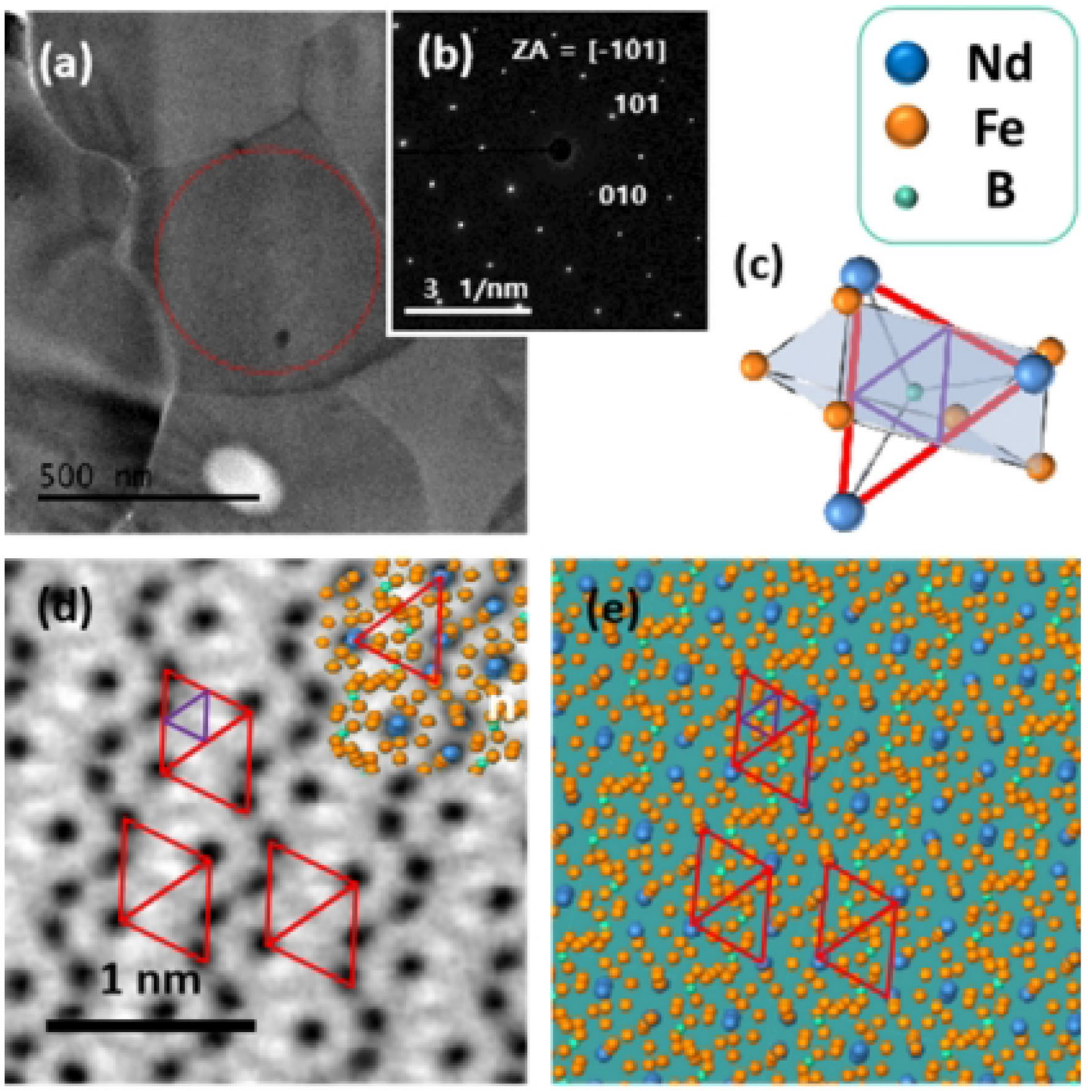
(**a**) TEM image of the Nd_1.5_Dy_0.5_Fe_14_B, (**b**) SAED pattern taken from the red circle in (**a**), (**c**) Nd_2_Fe_14_B trigonal prism in the [− 101] viewing zone axis, (**d**) high resolution BF-STEM image of Nd_1.5_Dy_0.5_Fe_14_B at [− 101] zone axis and (**e**) simulation of arrangement of the atoms by JEMS software.

To evaluate the crystallinity of the specimen, SAED patterns (Fig. 3b) of the marked area with red circle in the TEM image (Fig. 3a) were obtained. It was confirmed that the particles produced were single crystalline. It was deduced by SADP of strong diffraction maxima that each grain was completely single crystalline. Figure 3d shows the high resolution BF-STEM image of the Nd_1.5_Dy_0.5_Fe_14_B observed at the [− 101] zone axis and Fig. 3e represents the corresponding atomic arrangement simulated by JEMS software (P. Stadelmann, www.jems-saas.ch).

Both the BF-STEM image and simulated atomic arrangement showed the series of trigonal prism units. Figure 3c illustrates the arrangement of the atoms in the prism. Boron atom occupies the center of trigonal prisms surrounded with three nearest Fe atoms on top and the three Fe atoms at bottom. The triangular prism facets participate to form the complete tetragonal Nd_2_Fe_14_B lattice.

**Figure 4:**
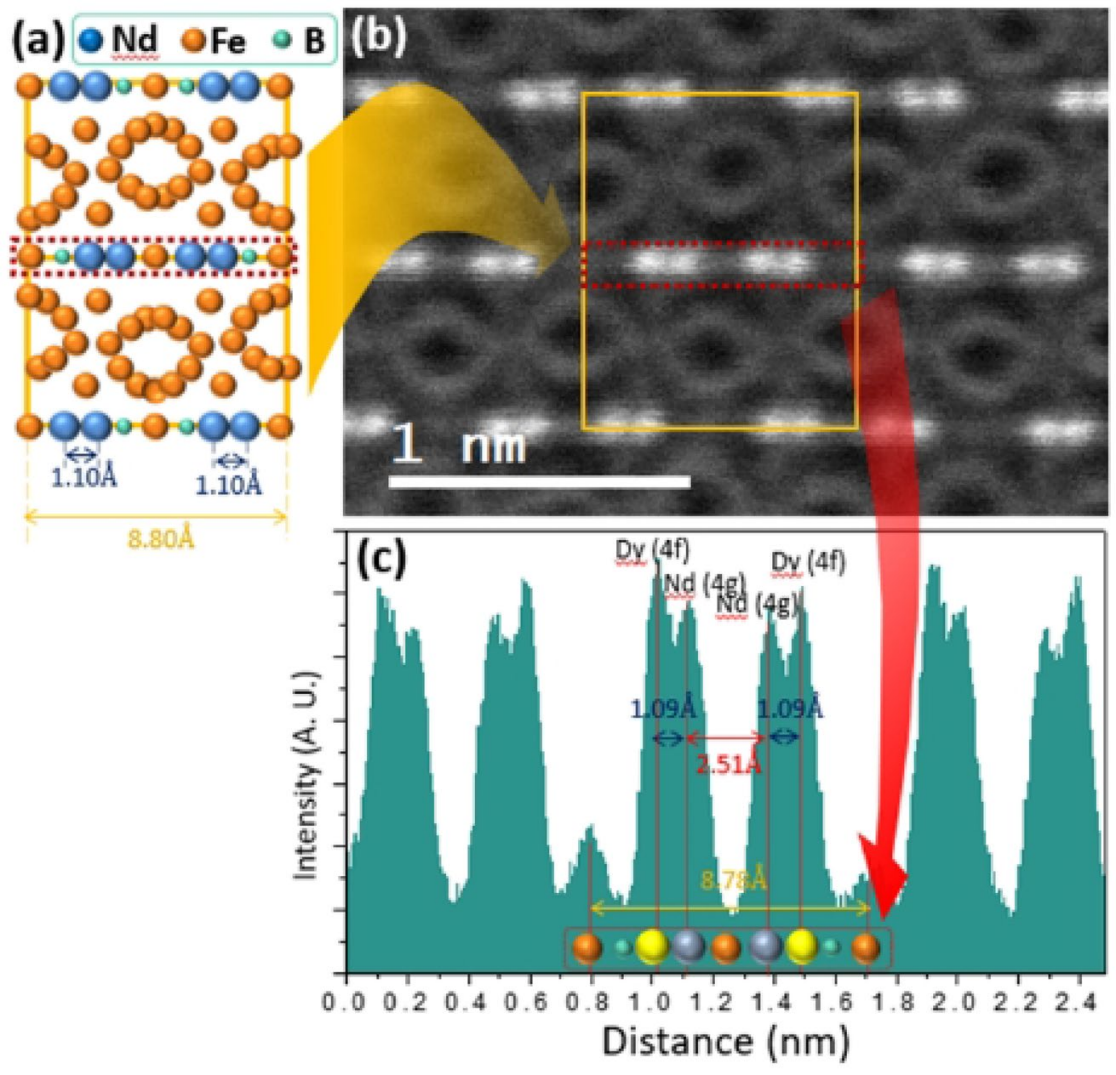
(**a**) Standard Nd_2_Fe_14_B unit cell with [100] zone axis (**b**) HADDF-STEM image of Nd_1.5_Dy_0.5_Fe_14_B at [100] zone axis, (**c**) intensity histogram for the atoms in the red dotted panel in (**b**).

To know the accurate site of the RE in the crystal lattice, HAADF-STEM image was taken at the [100] zone axis as shown in Fig. 4. At [100] zone axis, columns having “4f” site and “4 g” site of Nd/Dy can be clearly distinguished. In addition, there is no Fe atom overlapping at each Nd position because of different locations of Fe and RE at [100] zone axis. Peak intensity of the histogram increases with the average atomic number, the HAADF-STEM image can be used to distinguish the Dy and Nd, and their positions (“f” or “g” site). Figure 4b is HAADF-STEM image, which confirms the same arrangement of atoms as the standard Nd_2_Fe_14_B [100] zone axis (Fig. 3a). Intensity histogram for red dotted panel in Fig. 4b was acquired. It is observed that the intensity of atoms (Dy) at “4f” column is higher than that of the atom (Nd) at “4 g” column. Higher peak intensity confirms that the substitution site of Dy is “4f” site because the atomic number of Dy (66) is larger than that of Nd (60), which leads to the higher intensity as compared to the Nd. The ‘a’ value of Nd_1.5_Dy_0.5_Fe_14_B crystal lattice is 8.78 Å (Fig. 3c), which is well consistent with the standard Nd_1.5_Dy_0.5_Fe_14_B value.

**Figure 5:**
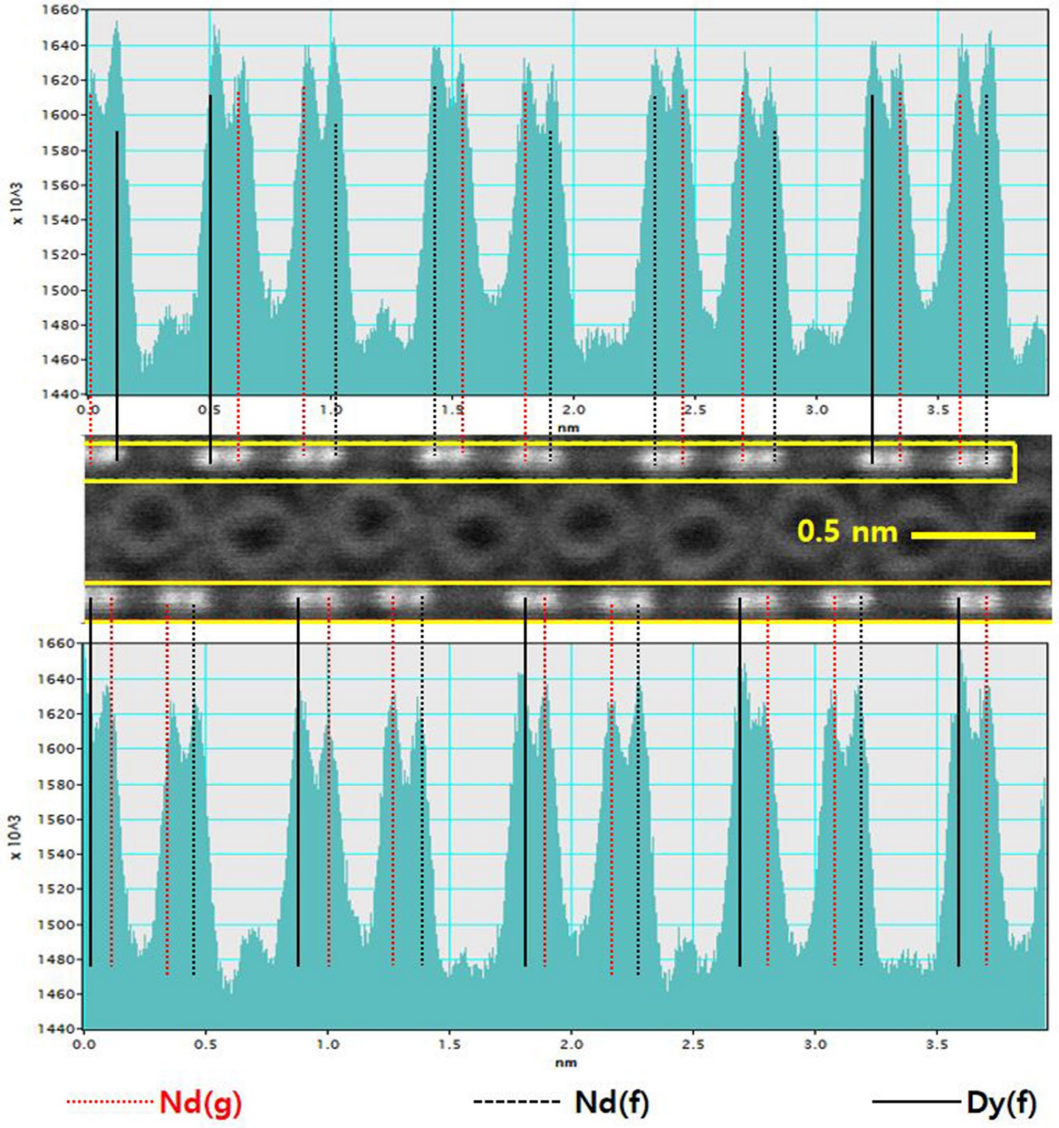
HADDF-STEM image of Nd_1.5_Dy_0.5_Fe_14_B at [100] zone axis, with intensity histogram. Light grey circles in the center indicate Fe atoms.

Standard Nd_2_Fe_14_B have ‘a’ and ‘b’ values of lattice parameters as 8.80 Å^30^. Standard distance of atomic column between “4f” site and “4 g” site of Nd_2_Fe_14_B is 1.1 Å. In this study, the obtained distance is 1.09 Å as shown in histogram Fig. 4c. This is well matched with theoretical value. A slight error is due to a noise induced by the fine drift of the sample or the poisson noise in the STEM. Hence, site preference for the Dy in Nd_2_Fe_14_B is proved to be “4f” as the previously theoretically reported by Liu et al.^21^. It was found that 100% “g” sites are occupied by the Nd (Fig. 5) and Dy was substituted only at “f” site.

**Figure 6:**
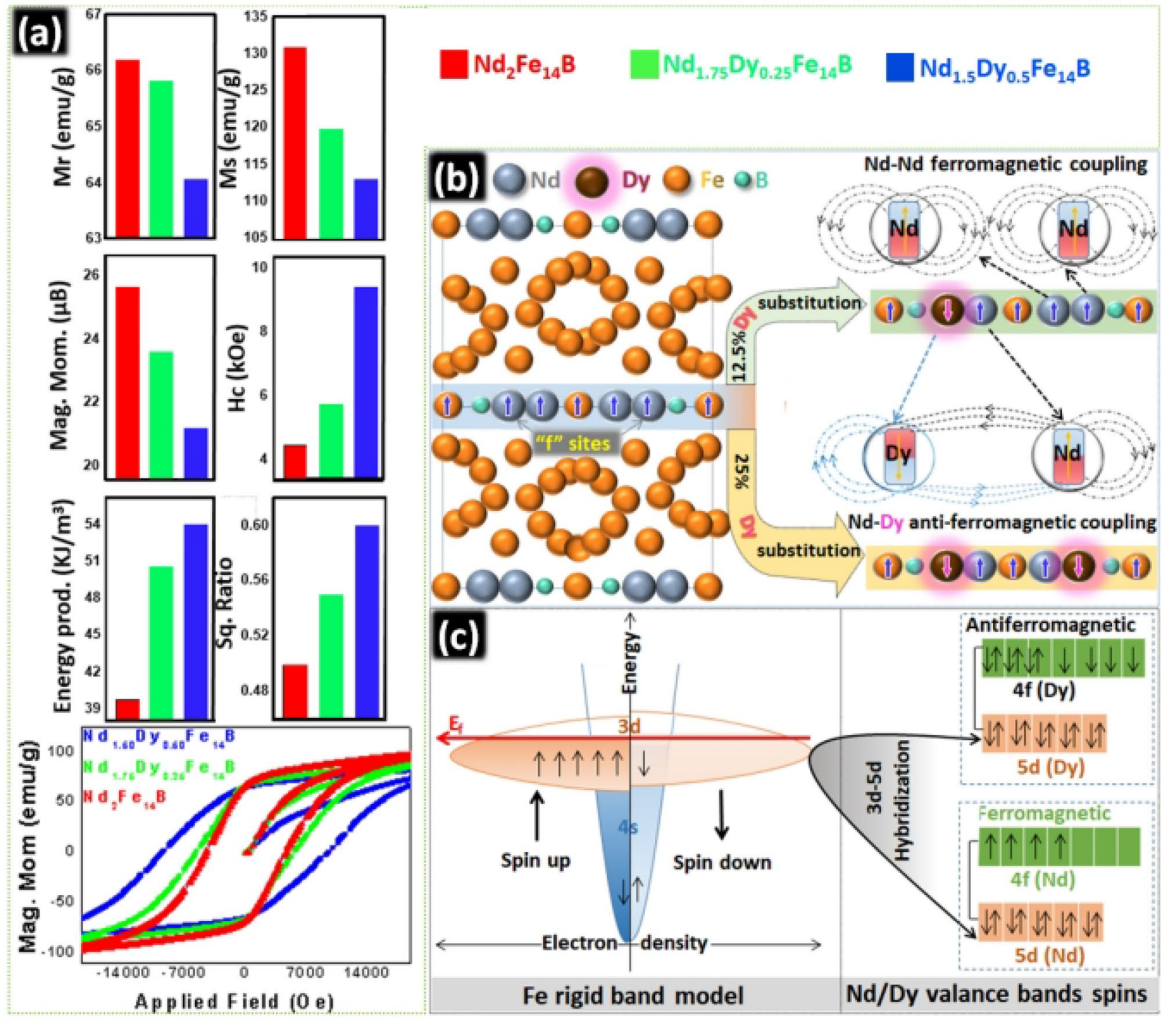
(**a**) M–H curves, Mr, Ms, Magnetic moment, Hc, energy density and squareness ratio of Nd_2_Fe_14_B, Nd_1.75_Dy_0.25_Fe_14_B and Nd_1.5_Dy_0.5_Fe_14_B particles. (**b**) Schematic illustration to explain the ferro and anti-ferromagnetic coupling between Nd, Dy and Fe (**c**) Explanation of coupling and hybridization between Nd, Dy and Fe.

Fe is ferromagnetic with electronic configuration of [Ar] 3d^6^ 4s^2^. This electronic configuration shows that it has eight valance electrons. Arrangement of the electrons in the relevant orbitals is shown in the rigid band model as Fig. 6c. Density of the electrons was taken on X-axis and energy was taken on Y-axis. E_f_ indicates the Fermi level of the rigid band. Energy level of 3-d electrons is similar to the 4 s electrons, hence, there is no movement of electrons between the 4s and 3d orbital. Four unpaired electrons will be in the spin up configuration. Presence of the unpaired electrons makes the Fe ferromagnetic.

Being completely filled, 5d orbitals in Nd or Dy do not play any role to determine the magnetic properties of Nd or Dy. However, Nd and Dy have unpaired electrons in the 4f orbitals, those impart the ferromagnetic character. Orbital magnetic moment (L) direction of the unpaired valance electrons in Dy is opposite to the Nd because 4f electrons in Dy are in spin down state. Nd has the unpaired electrons in the spin up direction, hence, they are ferromagnetic with the Fe.

Simultaneously Dy with the spin down configuration couples anti-ferromagnetically with Fe and Nd (Fig. 6c). 3d band in Fe may hybridize with the 5d orbital of the neighboring Nd and/or Dy. Schematic illustration of the exchange coupling and hybridization in the Nd_2_Fe_14_B and Nd_2−X_Dy_X_Fe_14_B is shown in Fig. 6c.

Magnetic moment of Nd_2−x_Dy_x_Fe_14_B have been strongly affected by Dy owing to its anti-ferromagnetic coupling with Fe and Nd. Individual values of magnetic moments of Nd_2_Fe_14_B, Nd_1.75_Dy_0.25_Fe_14_B and Nd_1.5_Dy_0.50_Fe_14_B, were determined as 25.50, 23.48, and 21.03 μB, respectively. Magnetic moments were determined by the the M_s_ values from M–H curves (Fig. 6a). Complete M-H curves with applied magnetic field range of − 9.5 to 9.5 T are provided in supplementary information as Fig. S-7. These experimentally determined values of magnetic moment are lower than the values by theoretical calculation because the theoretical parameter conditions are not fixed as the experimental ones^21^. For example, the theoretical calculation was based on the temperature at 5 K and all particles are single domain and un-oxidized. Dy coupled anti-ferromagnetically to Fe in the crystal lattice, resulting in the lower magnetic moment of Nd_2−x_Dy_x_Fe_14_B. The change of magnetic moment critically affected the coercivity. From the Fig. 6 the coercivity (Hc) values of Nd_2_Fe_14_B, Nd_1.75_Dy_0.25_Fe_14_B and Nd_1.5_Dy_0.50_Fe_14_B were determined as 4.58, 5.84 and 9.55 kOe, respectively. Nd_2−x_Dy_x_Fe_14_B showed the higher H_c_ due to stronger anisotropy energy and reduced magnetic moment. Additionally, Dy substituted particles have a smaller grain size as shown in SEM results. It is well known that coercivity increases as particle size gets smaller and approaches to the single domain size. The increasing order of coercivity as Nd_2_Fe_14_B < Nd_1.75_Dy_0.25_Fe_14_B < Nd_1.5_Dy_0.5_Fe_14_B and the decreasing order of magnetic moment as Nd_2_Fe_14_B > Nd_1.75_Dy_0.25_Fe_14_B > Nd_1.5_Dy_0.5_Fe_14_B were obtained from M–H curves.

Energy density or energy product is the amount of energy stored in the anisotropic Nd_2_Fe_14_B (or Nd_2−x_Dy_x_Fe_14_B) lattice because of arrangement of the atoms in the crystal. It is confirmed that the Dy substitutions results in the higher energy density. Energy densities for Nd_2_Fe_14_B, Nd_1.75_Dy_0.25_Fe_14_B and Nd_1.5_Dy_0.50_Fe_14_B were recorded as 39.71, 50.29 and 53.71 kJ/m^3^, respectively. M_r_ (emu/g), M_s_ (emu/g), squareness ratio (S_q_), magnetic moment (μB), coercivity (H_c_), and energy density values for the all Nd_2_Fe_14_B and Nd_2−x_Dy_x_Fe_14_B particles are shown in Fig. 6b, comparatively. M-H curve with S.I. units of coercivity (kA/m) is provided in supplementary information as Fig. S8.

**Figure 7:**
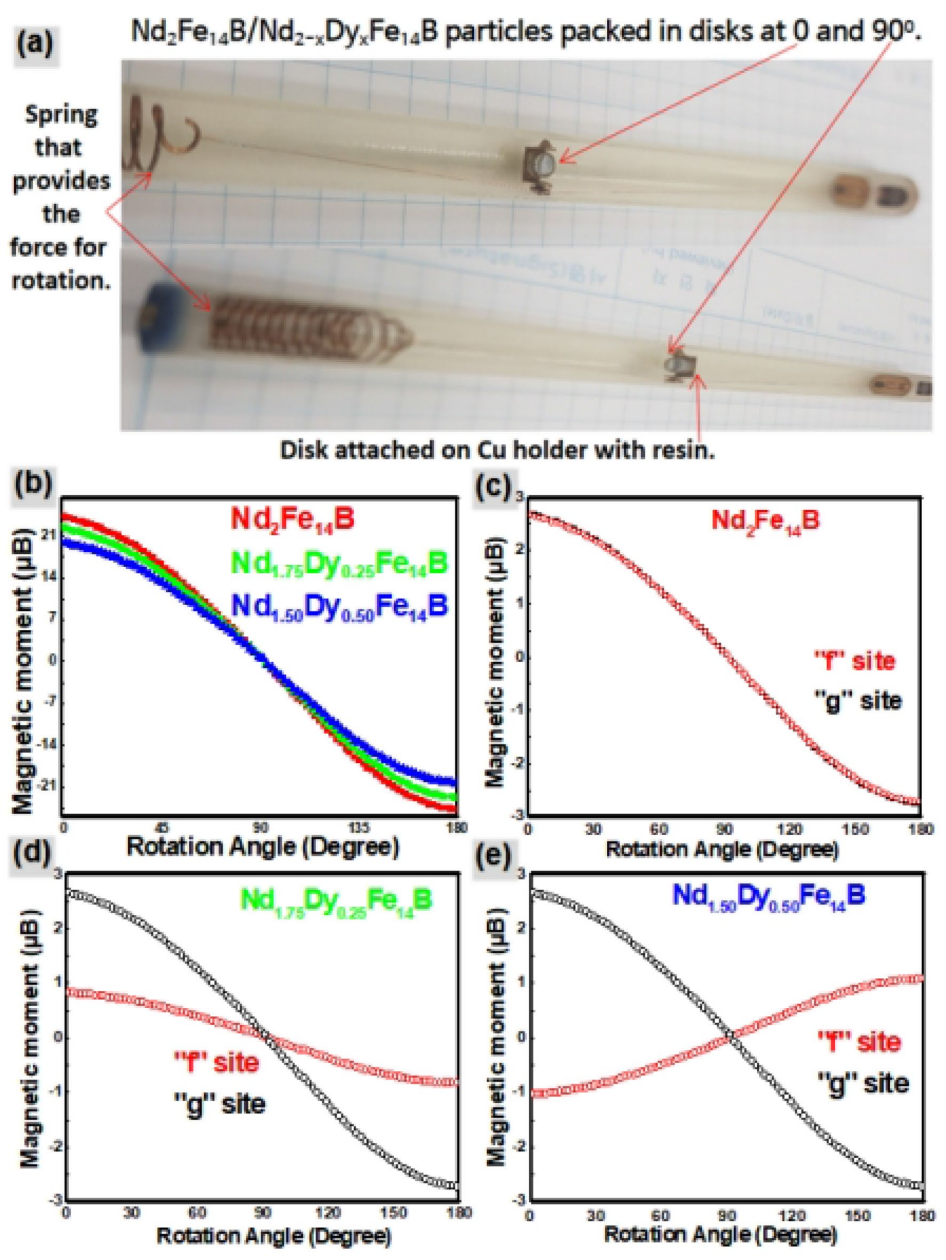
(**a**) Sample preparation for the measurement of magnetic anisotropy (**b**) Magnetic moment of Nd_2_Fe_14_B, Nd_1.75_Dy_0.25_Fe_14_B and Nd_1.5_Dy_0.5_Fe_14_B particles as a function of rotation angle. Magnetic moment at the “f” and “g” sites in (**c**) Nd_2_Fe_14_B (**d**) Nd_1.5_Dy_0.5_Fe_14_B (**e**) Nd_1.75_Dy_0.25_Fe_14_B as a function of rotation angle.

Nd_2_Fe_14_B and Nd_2−x_Dy_x_Fe_14_B are expected to be anisotropic, hence, magnetic moment and energy density are angular dependent magnetic properties. Closely packed particles of Nd_2_Fe_14_B and Nd_2−x_Dy_x_Fe_14_B were aligned at 5 T with easy direction of magnetization, then magnetic field was reduced to the 200 Oe. Thereafter, sample was rotated in the angle range of 0°–180°. Figure 7a explains the preparation of the sample for the measurement of magnetic anisotropy. During the measurement at MPMS, the magnetic particles were closely packed, which stopped the rotation of the particles at low applied magnetic field (200 Oe). All processes were performed at room temperature so that the effect of thermal energy was neglected.

Figure 7b shows the anisotropic character of the Nd_2_Fe_14_B and Nd_2−x_Dy_x_Fe_14_B. When magnetic particles were rotated in the range of 0°–180° at constant applied magnetic field of 200 Oe, magnetic moment of the particles was changed significantly. Nd_2_Fe_14_B and Nd_2−x_Dy_x_Fe_14_B have maximum magnetic moment along “c” crystal direction, with parallel/anti-parallel orientation to the applied field of (θ = 0°, 180°). This is because of the “c” crystal dimension is easy direction for magnetization. On the contrary, along “a and b” crystal direction magnetic moment approached to zero.

Maximum magnetic moment value (24.13 μB) of Nd_2_Fe_14_B obtained during rotation was lower than the magnetic moment values obtained from M–H curves (25.50 μB). Reduced value of magnetic moment was observed because of weak applied magnetic field (200 Oe) during the rotation of the magnetic particles. However, the trend of magnetic moment variation for both the Nd_2_Fe_14_B and Nd_2−x_Dy_x_Fe_14_B was same. Conclusively, substitution of Dy for Nd does not affect the anisotropic patterns of the crystal, however, value of the energy density can be changed. Figure S-8 explains the interaction of the applied magnetic field and the electron spin density of Nd_2_Fe_14_B during the rotation.

Magnetic moments of Nd_2_Fe_14_B, Nd_2−x_Dy_x_Fe_14_B and Nd were calculated theoretically by full potential linearized augmented plane wave method, as implemented in the Wien2k code. Individual magnetic moments of Nd and Dy atoms at different sites were also calculated theoretically by this method. Theoretically calculated magnetic moment values for Nd_2_Fe_14_B, Nd (f) and Nd (g) were 30.20, 3.32 3.30 μB, respectively. Experimentally determined values for the Nd_2_Fe_14_B, Nd (f) Nd (g) were 24.3, 2.69, 2.67 μB, respectively. Details of experimental and theoretical calculations are provided in the “supplementary information”.

Theoretically Nd always occupies the “g” site in Nd_2_Fe_14_B, Nd_1.75_Dy_0.25_Fe_14_B and Nd_1.5_Dy_0.50_Fe_14_B^19–21^. This is also confirmed in this work (Fig. 4). Hence in case of Nd_1.75_Dy_0.25_Fe_14_B and Nd_1.5_Dy_0.50_Fe_14_B there is negligible change in the magnetic moment on the “g” site after substitution. In Nd_2_Fe_14_B formula unit, Nd is distributed equally among 50% “f” and 50% “g” sites. Magnetic moment on “f” and “g” sites of in Nd_2_Fe_14_B is determined as 2.69 and 2.67 μB, respectively.

In Nd_1.75_Dy_0.25_Fe_14_B and Nd_1.5_Dy_0.50_Fe_14_B, Nd occupies 75% and 50% “f” sites, simultaneously Dy occupies leftover 25% and 50% “f” sites respectively. Hence magnetic moment on one “f” sites in Nd_1.75_Dy_0.25_Fe_14_B and Nd_1.5_Dy_0.50_Fe_14_B was determined as 0.82 and − 1.05 μB, respectively. Dy, which has almost double magnetic moment (5.34 μB) as compared to the Nd (2.45 μB), reduces the magnetic moment very effectively after the substitution. Reduction of magnetic moment of in the Nd_2−x_Dy_x_Fe_14_B formula unit is actually the reduction of the magnetic moment of the “f” site.

After obtaining the experimental values of the magnetic moments of the Nd_2_Fe_14_B, Nd (f) and Nd (g), anisotropic behavior of “f” and “g” sites was studied. Figure 7a explains the sample preparation for the measurement of magnetic anisotropy. Nd_2_Fe_14_B, and Nd_1.75_Dy_0.25_Fe_14_B particles were compressed in the plastic discs and then attached to the rotating plate with the resin. Wires and springs used in the apparatus are made of Cu, which is non-magnetic. When these samples were kept in the MPMS they were rotated by application of force on the spring. Magnetic moment values of the “f” and “g” sites in Nd_2_Fe_14_B and Nd_2−x_Dy_x_Fe_14_B at various angles of rotation are given in the Table 1. Figure 7b graphically illustrates that the magnetic moment of Nd_2_Fe_14_B and Nd_2−x_Dy_x_Fe_14_B as the function of rotating angle in the constant magnetic field. Figure 7c–e shows the variation of individual magnetic moments of “f” and “g” sites as the function of rotating angle.

**Table 1:** Magnetic moment values of Nd_2_Fe_14_B and Nd_2−x_Dy_x_Fe_14_B at “f” and “g” sites at rotating angle of 0° and 180°.

Substitution sites	Magnetic moment (μB)		
	Nd_2_Fe_14_B	Nd_1.75_Dy_.25_Fe_14_B	Nd_1.50_Dy_.50_Fe_14_B
f sites (0°)	2.69	0.82	− 1.05
f sites (180°)	− 2.71	− 0.85	1.06
g sites (0°)	2.67	2.67	2.67
g sites (180°)	− 2.71	− 2.71	− 2.71

## Conclusions

Nd_2_Fe_14_B and Nd_2−x_Dy_x_Fe_14_B (x = 0.25, 0.50) particles were successfully prepared by the modified co-precipitation method followed by reduction–diffusion process. Micro-structure analysis of the composition and distribution of elements confirmed the homogeneous distribution of Dy atoms in the crystal lattice of Nd_2_Fe_14_B and Dy substitution at “4-f” site. Orbital magnetic moment (L) direction of the unpaired valance electrons of Dy was opposite to the Fe and Nd which resulted in the anti-ferromagnetic coupling between them. Nd substitution with Dy on “f” site reduced the magnetic moment of Nd_2_Dy_x_Fe_14_B due to anti-ferromagnetic coupling with Nd and Fe, but enhanced the energy density, squareness ratio and coercivity. Furthermore, it was found that Nd_2_Fe_14_B and Nd_2−x_Dy_x_Fe_14_B particles have maximum magnetic moment when they are aligned parallel or anti-parallel to the applied magnetic field and have the minimum energy density when they are rotated perpendicular to the applied magnetic field. Conclusively, “f” site substitution of Nd with Dy in Nd_2_Fe_14_B did not change the anisotropic patterns of Nd_2−x_Dy_x_Fe_14_B.

## Supplementary Information

Supplementary Information.
