# Supplementary material for: Determination of Dy substitution site in Nd2−xDyxFe14B by HAADF-STEM and illustration of magnetic anisotropy of “g” and “f” sites, before and after substitution
Source: Sci Rep. 2021 Mar 18;11:6347. doi: 10.1038/s41598-021-85713-5 (PMC7973419; doi:10.1038/s41598-021-85713-5)
Supplement: Supplementary file 1 — Supplementary Information. [file 41598_2021_85713_MOESM1_ESM.docx]

Determination of Dy Substitution Site in Nd_2-x_Dy_x_Fe_14_B by HAADF-STEM and Illustration of Magnetic Anisotropy of “g” and “f” sites, Before and After Substitution

*Syed K. Haider^a,b,c^‡, Min-Chul Kang^d^‡, Jisang Hong^e^, Young Soo Kang^c*^, Cheol-Woong Yang^d*^, Dongsoo Kim^a,b,*^*

^a^ Convergence research center for development of mineral resources, Korea Institute of Geoscience and Mineral Resources, Daejeon 34132, South Korea.

^b^ Powder & Ceramics Division, Korea Institute of Materials Science, Changwon, Gyeongnam 51508, South Korea.

^c^ Department of Chemistry, Sogang University, 35, Baekbeomro, Mapogu, Seoul, 04107, South Korea.

^d^ School of Advanced Materials Science and Engineering, Sungkyunkwan University, Suwon 16419, Korea.

*^e^* Department of Physics, Pukyong National University, Busan 48513, Korea.

*Corresponding authors.

‡These authors contributed equally to this work.

[yskang@sogang.ac.kr](mailto:yskang@sogang.ac.kr)

**Synthesis of Nd_2_Fe_14_B, and Nd_2-x_Dy_x_Fe_14_B magnetic particles**

Co-precipitation method in this work was modified to enhance the magnetic properties based on the previous the work of *Ma^1^* and *Palaka et al.^2^* Both of the previous works used 3.5 M NaOH for co-precipitation (same as this work) but during the co-precipitation pH was raised up to 10. In this work during the co-precipitation, pH was raised up to 13 and following changes were observed,

1. In our work at pH 10, percentage yield of co-precipitation product was 92% and at pH 13, it increased up to 99%.
2. After co-precipitation at pH 10, some of the precipitates were suspended in the solution and it took more than 2 hours for precipitates to settle down. But after co-precipitation at pH 13, it took 25 minutes for precipitates to settled down. When precipitates settle down completely, more than 80% of the byproduct (solution of the NaCl and NaOH dissolved in the water) can be removed just by decanting, without any centrifugation.
3. In order to remove the by-products of co-precipitation *Ma et al.* and *Palaka et al.^1,2^* used centrifugation at 8000 rpm for 5 minutes. In this work after the co-precipitation centrifugation at 4000 rpm for 45 seconds removed all the byproducts.

For the synthesis Nd_2_Fe_14_B, and Nd_2-x_Dy_x_Fe_14_B, it is important to bring the oxides particles of the RE, Fe and B close to each other and they should be homogeneously mixed. The particle size distribution of the precursors is also important. To get a homogeneous and even particle-size mixture of precursors, the co-precipitation method was used. For the sake of convenience, we shall write both Nd and Dy as RE in supporting information. Chlorides of REs and Fe were changed to hydroxides using NaOH.

# FeCl_3_ + 3NaOH
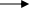
 Fe(OH)_3_ + 3NaCl (1)

# RECl_3_ + 3NaOH
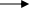
 RE(OH)_3_ + 3NaCl (2)

Initial pH of the chloride solution was 2.61 and all RE and Fe chlorides were converted to hydroxides at pH 7 but pH was raised to 13 because this gave optimum result with 99% yield.

Standard reduction potential for the Fe^3+^ is higher, as compared to the REs in the solution, those have standard reduction potential below -2 V. RE were stable most positive ion and was precipitated at pH around 7, however all Fe were precipitated before RE in the form of Fe(OH)_3_.6H_2_O and RE_l-x_ Fe_x_(OH)_3_. RE_l-x_Fe_x_ (OH)_3_ and Fe(OH)_3_.6H_2_O were changed to the REFeO_3_ and Fe_3_O_4_ respectively by annealing at 700 ^o^C in the presence of air. Oxides obtained in this way had an average particle size of 30 nm (Figure S 2). EDX analysis confirmed the homogeneous distribution of the RE and metal oxide particles. This oxide mixture was mixed with CaH_2_ and boric acid, and then pressed into pellet form. This pellet was annealed at 1000 °C and the reduction-diffusion of the oxides was completed. Possible chemical reactions during the reduction diffusion process are given below with help of the reaction scheme proposed by *Haider et al.1*^3^. At first boric acid is decomposed to boron oxide. This is a two-step reaction and is very rapid in the temperature range of 150–170 °C.

# H_3_BO_3_
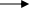
 HBO_3_ + H_2_O (3)

# 2HBO_2_
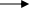
 B_2_O_3_ + H_2_O (4)

Next reaction is the reduction of Fe_2_O_3_ and B_2_O_3_. These reactions take place around 300 °C.

Fe_2_O_3_ + 3CaH_2_
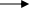
 2Fe + CaO + 3H_2_ (5)

B_2_O_3_ + CaH_2_
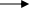
 2B + CaO + 3H_2_  (6)

Next is the reduction of REFeO_3_ and RE_2_O_3_. When the RE is Nd, these reactions take place at 620 °C.

RE_2_O_3_ + 3CaH_2_
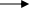
 2REH_2_ + 3CaO + H_2_ (7)

REFeO_3_ + 3CaH_2_
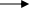
 REH_2_ + Fe + 3CaO + 2H_2_ (8)

Last step is the diffusion reaction to produce RE_2_Fe_14_B. When RE is Nd, these reactions usually take place at 692 °C. 10% extra Nd was added in the start of each experiment because some Nd is lost during the R-D process.

REH_2_ + 17Fe
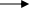
 RE2Fe_17_ + 2H_2_  (9)

RE_2_Fe_17_ + B
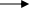
 RE_2_Fe_14_B + 3Fe (10)

2REH_2_ + 14Fe + B
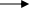
 RE_2_Fe_14_B + 2H_2_ (11)

The overall reaction in the process can be described as following (Eq. 12).

2H_3_BO_3_+13Fe_2_O_3_+RE_2_O_3_+2REFeO_3_ + 53CaH_2_
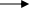
2RE_2_Fe_14_B + 55H_2_ + 53CaO + H_2_O (12)

**Computational calculations for the magnetic moment**

Magnetic moments of Nd_2_Fe_14_B and Nd_2-x_Dy_x_Fe_14_B, Nd were theoretically calculated by full potential linearized augmented plane wave method in our previous work.^5^ Full potential linearized augmented plane wave method, as implemented in the Wien2k code was used to study the electronic and the magnetic properties of Nd_2_Fe_14_B. In our calculations experimental lattice constants of Nd_2_Fe_14_B unit cell were opted as, a = b = 8.805 and c = 12.206. The local spin density approximation with Hubbard-type on-site Coulomb potential U (LDA + U) was opted as the exchange and correlation potential. In order to expand the charge, potential and wave functions in the muffiin-tin region, spherical harmonics with *l_max_* = 10 were used. Charge density was Fourier expanded up to G*_max_* = 12, in the interstitial region. To expand the wave function in the interstitial region cut-off parameter of R*_mt_*K*_max_* = 9 was used, where R*_mt_* is the smallest atomic sphere radius in the unit cell. Maximum value of the reciprocal lattice vector used in the plane wave expansion was K*_max_*. Value of muffin-tin radii for Nd, Fe, and B were considered as 2.5, 2.08 and 1.84 a.u. respectively and employed 5 × 5 × 3 Monkhorst-Pack mesh scheme.

Simultaneously magnetic moments of Nd and Dy at different sites were also calculated theoretically by this method.^5^ Theoretically calculated magnetic moment values for Nd_2_Fe_14_B, Nd (f) Nd (g) were taken from the Table1 of ref 27. Experimentally calculated values (24.3 μB) of the total magnetic moment were lower as compared to the ref 27 (30.32 μB) hence. When the 30.32 μB was multiplied with the factor 0.8014, 24.3 μB was obtained which is the actual value obtained from the experiment. Magnetic moments of Nd (f) Nd (g) were also multiplied with the 0.8014 and experimental values of the magnetic moments were obtained. Magnetic moment of the Dy was calculated by the total difference of the magnetic moment of Nd_2_Fe_14_B and Nd_x-2_Dy_x_Fe_14_B.

**Figures and Figure Captions**


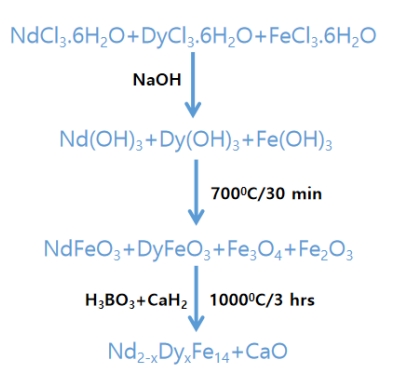


Figure. S 1**.** Schematic illustration on the process for the synthesis of Nd_2_Fe_14_B and Nd_x-2_Dy_x_Fe_14_B particles.


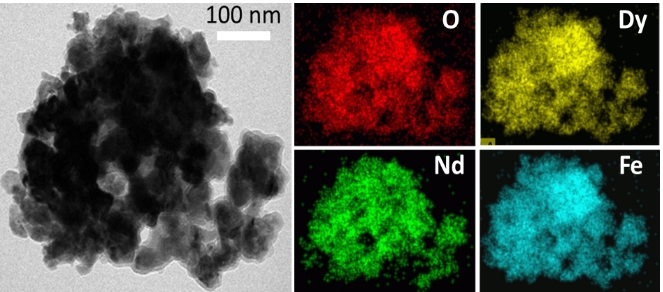


Figure. S 2. TEM and TEM-EDS mapping images for the oxide intermediates after annealing of hydroxide precipitates.


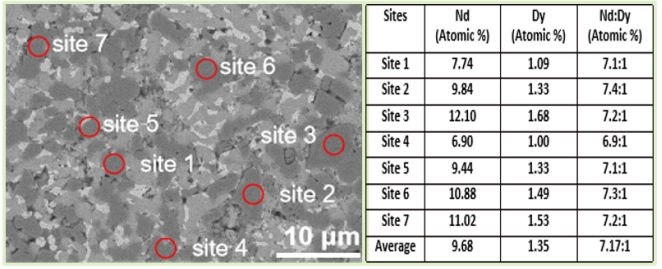


Figure. S 3. Atomic percentage and ratio of Nd and Dy at various sites in Nd_17.5_Dy_0.25_Fe_14_B measured by SEM-EDS point analysis.


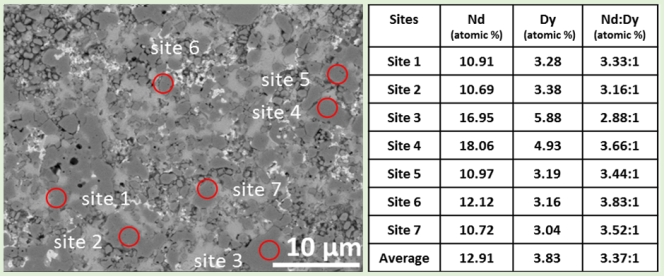


Figure. S 4. Atomic percentage and ratio of Nd and Dy at various sites in Nd_1.5_Dy_0.5_Fe_14_B measured by SEM-EDS point analysis.


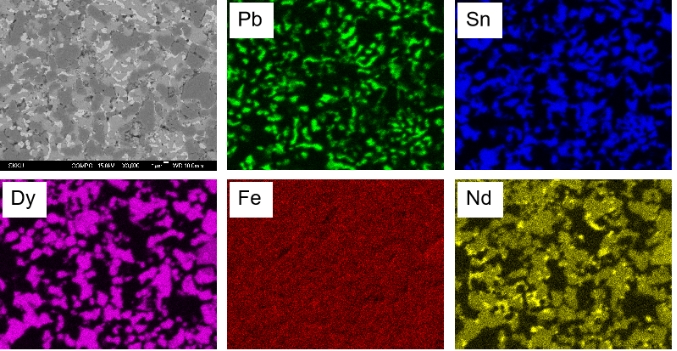


Figure. S 5 SEM -EDS images of Nd_1.75_Dy_0.25_Fe_14_B.


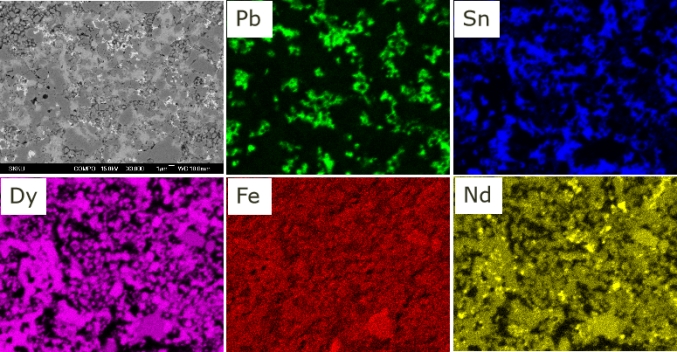


Figure. S 6. SEM -EDS images of Nd_1.5_Dy_0.5_Fe_14_B.


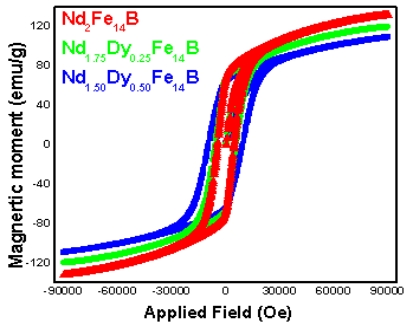


Figure. S 7. M-H curves for Nd_2_Fe_14_B, Nd_1.75_Dy_0.25_Fe_14_B a Nd_1.5_Dy_0.5_Fe_14_B magnetic particles.

Figure. S 8. M-H curves for Nd_2_Fe_14_B, Nd_1.75_Dy_0.25_Fe_14_B a Nd_1.5_Dy_0.5_Fe_14_B magnetic particles with S.I. units.

**References**

(1) Z. Ma, Z., Zhang, T. & Jiang, C. [A facile synthesis of high performance SmCo](https://www.sciencedirect.com/science/article/pii/S1385894714016064)_[5](https://www.sciencedirect.com/science/article/pii/S1385894714016064)_ [nanoparticles](https://www.sciencedirect.com/science/article/pii/S1385894714016064) *Chem. Eng. J.,* 2015, 264, 610–616. [https://doi.org/10.1016/j.cej.2014.11.138](https://doi.org/10.1016/j.cej.2014.11.138)

(2) Palaka, S.,Yue, M., Ma, Z., Li, C,, Li, H., Xu, H. & Cong, L. A facile chemical synthesis of PrCo_5_ particles with high performance. *J. Alloy. Comd.,* 2020, 812, 151674. [https://doi.org/10.1016/j.jallcom.2019.151674](https://doi.org/10.1016/j.jallcom.2019.151674)

(3) Syed, K. H., Jin-Young L., Dongsoo, K. & Kang, Y.S. Eco-Friendly Facile Three-Step Recycling Method of (Nd-RE)_2_Fe_14_B Magnet Sludge and Enhancement of (BH)max by Ball Milling in Ethanol *ACS Sustainable Chem. Eng.,* 2020, 8, 8156–8163. [https://doi.org/10.1021/acssuschemeng.0c00584](https://doi.org/10.1021/acssuschemeng.0c00584)

(4) Imran, K. & Jisang, H. Site Preferences for La and Pr in Nd_2_Fe_14_B Permanent Magnet: A First Principles Study. *J. Kor. Phys. Soci.*, 2016, 69, 1564-1570. 10.3938/jkps.69.1564

(5) Khan, I. & Hong, J. Electronic Structure and Magnetic Properties of Nd_2_Fe_14_B. *J. Kor. Phys. Soc.* 2016, 68, 1409-1414. DOI: 10.3938/jkps.68.1409
